# Vapour-Induced Liquid Crystallinity and Self-Recovery Mechanochromism of Helical Block Copolymer

**DOI:** 10.1038/s41598-017-03948-7

**Published:** 2017-06-21

**Authors:** Hiroki Hayashi, Tomokazu Iseki, Shigeki Nimori, Hiromasa Goto

**Affiliations:** 1 Division of Materials Science, Faculty of Pure and Applied Sciences, University of Tsukuba, Tsukuba, Ibaraki 305-8573 Japan; 2 Tsukuba Magnet Laboratory, National Institute for Materials Science (NIMS) Sakura 3-13, Tsukuba, Ibaraki 305-0003 Japan

## Abstract

New molecular design of conjugated polymer that possess high sensitivity to vapour and self-recovering property against pressure is proposed. We synthesised a rod-rod diblock copolymer, poly(3-((3*S*)-3,7-dimethyl-octyl)-thiophene)-block-poly(4-octyl phenylisocyanide) (**PTh-** ***b*** **-PPI**), composed of a π-conjugated polymer and a rod-type helical coiled polymer. Introduction of **PPI** block in the block copolymer architecture enabled **PTh-** ***b*** **-PPI** film to exhibit solid-to-liquid crystal phase transition by exposure to chloroform vapour, accompanied with colour change (purple-to-yellow), which is the first report on a new phenomenon of “vapour-induced liquid crystallinity”. In addition, **PTh-** ***b*** **-PPI** film showed colour change (purple-to-vermillion) during mechanical shearing, and spontaneously recovered under ambient conditions. We concluded that rod-type helical coiled polymer **PPI** block performs crucial roles as intrinsically vapour-induced liquid crystallinity and self-reassembling property in the architecture of **PTh-** ***b*** **-PPI**.

## Introduction

Stimuli-responsive materials have been attracted substantial attention for a wide variety of potential applications such as sensors^1^, drug delivery systems^2^ and actuators^3^. Responsiveness to external stimuli, for example heat, light, electrical and magnetic field, vapour and pressure, involves changes of molecular conformation and packing structure of molecules that converts physical signals into optical, electrical, mechanical and thermal signals^4^.

One of the strategies to create stimuli-responsive materials is the incorporation of liquid crystal (LC) component in the molecular structure. LC is one of the self-organized soft materials originating from weak interactions of van der Waals force and the excluded volume effect^5^. LC materials spontaneously form organized structures and possess dynamic properties against external stimuli such as shear-stress, electrical and magnetic field. With these advantages, the materials incorporated with well-designed LC moiety into the molecular structure show mechanical-induced phase transition^6, 7^, and form spontaneous ordered structures with anisotropic functionalities^8, 9^ and photo- and magnetic field-assisted macroscopic orientations^10, 11^.

Molecular design of conjugated polymers (CPs) with high stimuli-responsiveness is challenging task. Because CPs possess strong π-π interaction between polymer chains in aggregation state, the film hardly shows responsiveness to external stimuli. To control conformation of the CP backbones, modifications of side chain have been carried out, which provide improvement of solubility, promotion for self-organization with higher order, and suppression of inter-chain interaction between the CP backbones^12–14^. However, it is still challenging to create CP films with high sensitivities to weak external stimuli, such as vapour and low pressure. CPs are attractive systems since they have π-electrons delocalized over the polymer backbone that are origins of electronic, optical and magnetic properties. In this context, “soft CPs” with high sensitivity to the above stimuli is greatly desired for future smart polymers. If such small stimuli are converted to the dynamic conformational change of the CPs, the signals are amplified, resulting in the drastic changes in colour, conductivity and electronic properties.

**Figure 1:**
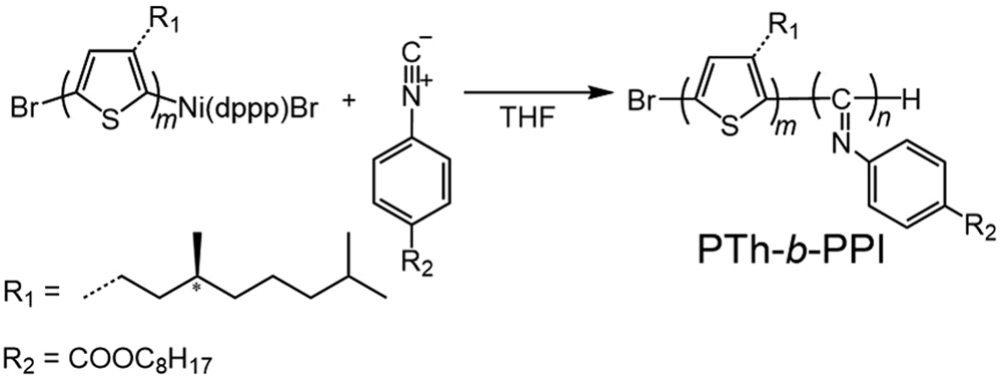
Molecular design. Synthetic route for **PTh-** ***b*** **-PPI** through Grignard metathesis (GRIM) reaction in one-pot. dppp = 1,3-Bis(diphenylphosphino)propane. THF = Tetrahydrofuran.

In this study, based on the recent advancement of supramolecular chemistry, we strategically designed block copolymer, poly(3-((3S)-3,7-dimethyl-octyl)-thiophene)-block-poly(4-octyl phenylisocyanide) (**PTh-** ***b*** **-PPI**), composed of a CP and a rod-type helical coiled polymer as shown in Fig. 1. Polythiophene is one of CPs and exhibits colour changes in visible range originating from the conformational changes in the polymer backbone between the planar state (purple) and the twisted state (yellow)^15^. The conformation of polythiophene that has chiral side chain has also been investigated, and it is considered to form helical packing of predominantly planar chains in the aggregation state driven by strong π-π interaction^16^. Introducing chiral side chain into polythiophene block also allows us to investigate the aggregation process in detail since we can track its process using circular dichroism (CD) spectroscopy. Therefore, we expect that the poly(3-((3S)-3,7-dimethyl-octyl)-thiophene (**PTh**) block can act as a colour-changing chiral chromophore in visible range by external stimuli. On the other hand, polyphenylisocyanide form rigid rod-like helical backbone. Polyphenylisocyanide with bulky side chains forms a rigid helical backbone not only in the solid state but also in solution. Because of its anisotropic rod-shaped structure, the polyphenylisocyanide segment can serve as a mesogen for liquid crystal superstructure in concentrated solution^17^. Recently, polyphenylisocyanide can also be used as an alignment medium to measure residual dipolar coupling in NMR studies^18^ since polyphenylisocyanide has magnetically anisotropic phenyl rings as side chains oriented in the peripheral position. Therefore, we envisioned that the poly(4-octyl phenylisocyanide) (**PPI**) unique structural motif has potential to be a novel responsive building block in block copolymers, which provides LC-like self-assembling and magnetic-field responsiveness.

Recently, Wu and Bielawski first reported the one-pot synthesis of polythiophene-block-polyphenylisocyanide, consisting of poly(3-hexylthiophene) and poly(decyl 4-phenylisocyanide)^19^. They have reported on a variety of morphologies of polythiophene-block-polyphenylisocyanide derivatives that form nanofibril, micelle and vesicle in a mixture of good and poor solvents and its pH-responsiveness^20–23^.

We coincidentally found potentially important phenomena of **PTh-** ***b*** **-PPI** film: vapour-induced liquid crystallinity and self-recovering mechanochromism. We found out **PPI** block performs crucial roles in the block copolymer, which are intrisically vapour-induced liquid crystallinity and self-reassembling property. **PTh-** ***b*** **-PPI** film possesses the softness and flexibility enough to show high sensitivity to solvent vapours and shear stresses.

## Results and Discussion

### Synthesis and characterisation of PTh-*b*-PPI

**Figure 2:**
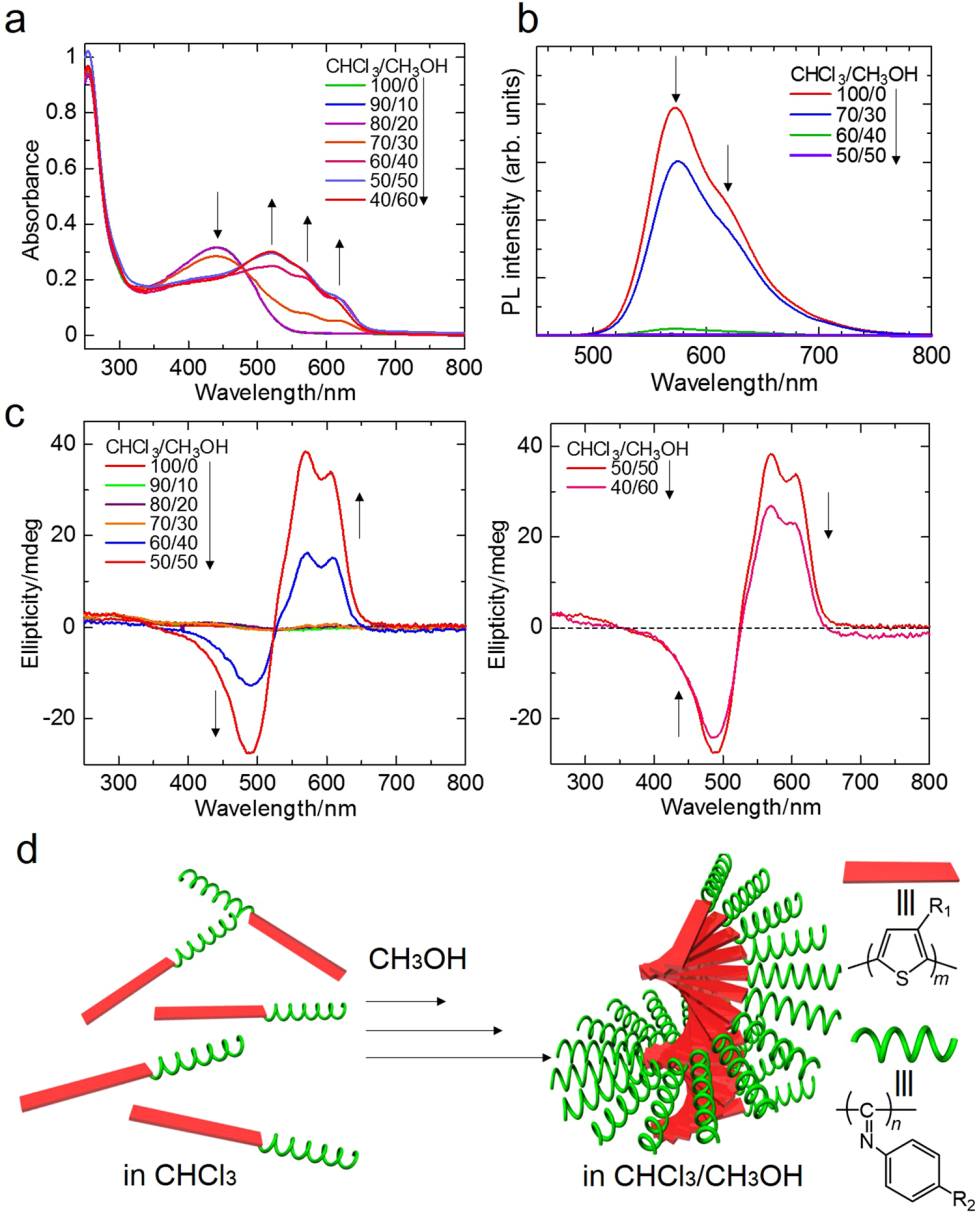
Chiral aggregation process of PTh-*b*-PPI in solution driven by interaction of PTh blocks. (**a**) UV-vis absorption spectra, (**b**) Photoluminescence (PL) spectra (λ_ex_ = 420 nm) and (**c**) Circular dichroism (CD) spectra of PTh-*b*-PPI in CHCl_3_/CH_3_OH solution (0.02 mg/ml) in various ratios. In high ratios of methanol, the spectra show film-like aggregation state. (**d**) Schematic illustration of PTh-*b*-PPI chiral aggregation in good/poor solvent mixture.

**PTh-** ***b*** **-PPI** was synthesised according to the previously reported literature using the Grignard metathesis (GRIM) reaction^19, 24, 25^. First, we prepared Ni-terminated **PTh** macro-initiator from 2,5-dibromothiophene in a flask, then **PPI** monomer was added. When the polymerisation ceased, the resultant polymer was washed with a large volume of methanol and collected by filtration. Gel-permeation chromatography (GPC) showed a number average molecular weight *M*_n_ = 16000 and a polydispersity *PDI* = 4.5. The *PDI* value was relatively high because the GRIM reaction usually proceeds in living fashion. The high value of PDI is probably caused by the broad polydispersity nature of Ni-teriminated **PTh** macroinitiator as reported in the literature^26^. **PTh-** ***b*** **-PPI** was thoroughly characterised by NMR, IR, UV, CD, and PL (Figures S1 and S2, Fig. 2a,b,c) and all the basic properties were well in accordance with those previously reported for polythiophene-*block*-polyphenylisocyanide derivatives^19–23^.

We first investigated the aggregation formation of **PTh-** ***b*** **-PPI** in good/poor solvent-mixture. This characterisation gives us the insights of film formation process and the interactions of **PTh-** ***b*** **-PPI**. In UV-vis spectra (Fig. 2a), **PTh-** ***b*** **-PPI** in chloroform shows absorption maxima at 255 nm (mainly from the **PPI** block) and 441 nm (derived from twisted state of the **PTh** block). Addition of methanol to the polymer solution decreases the absorption peak at 411 nm, and new signals appear near 520, 570, and 620 nm. This large red-shift indicates conformational change of the **PTh** block into *J*-aggregation states with planar main-chain conformation. The spectra strongly support formation of **PTh-** ***b*** **-PPI** aggregation driven by π-π interaction between the **PTh** blocks^26^. PL spectra (Fig. 2b) also reveals that the chloroform solution state **PTh-** ***b*** **-PPI** shows luminescence at 572 nm with a shoulder peak at ~630 nm. The PL signal is derived from twisted **PTh** blocks. As methanol is increased in the solution the PL intensity decreases, indicating formation of aggregation of **PTh** blocks. Furthermore, the chiroptical properties of **PTh-** ***b*** **-PPI** were investigated by CD spectroscopy (Fig. 2c). The **PTh-** ***b*** **-PPI** in chloroform shows no CD signal, indicating random conformation of **PTh-** ***b*** **-PPI**. In the chloroform/methanol (40/60 v/v), the CD signals appeared at 605, 570 nm (positive) and 490 nm (negative). Additionally, the wavelength at the cross section from positive to negative (at 523 nm) corresponds to the maximum absorption wavelength of **PTh-** ***b*** **-PPI** (at ~520 nm), indicating right-handed helical aggregation of PTh chromophores^27^. In high ratios of methanol, the CD intensity decreases because of the precipitation. These optical measurements suggest that *J*-aggregation of **PTh-** ***b*** **-PPI** is driven by π-π interaction of **PTh** blocks.

### Vapour-induced liquid crystallinity of PTh-*b*-PPI

**Figure 3:**
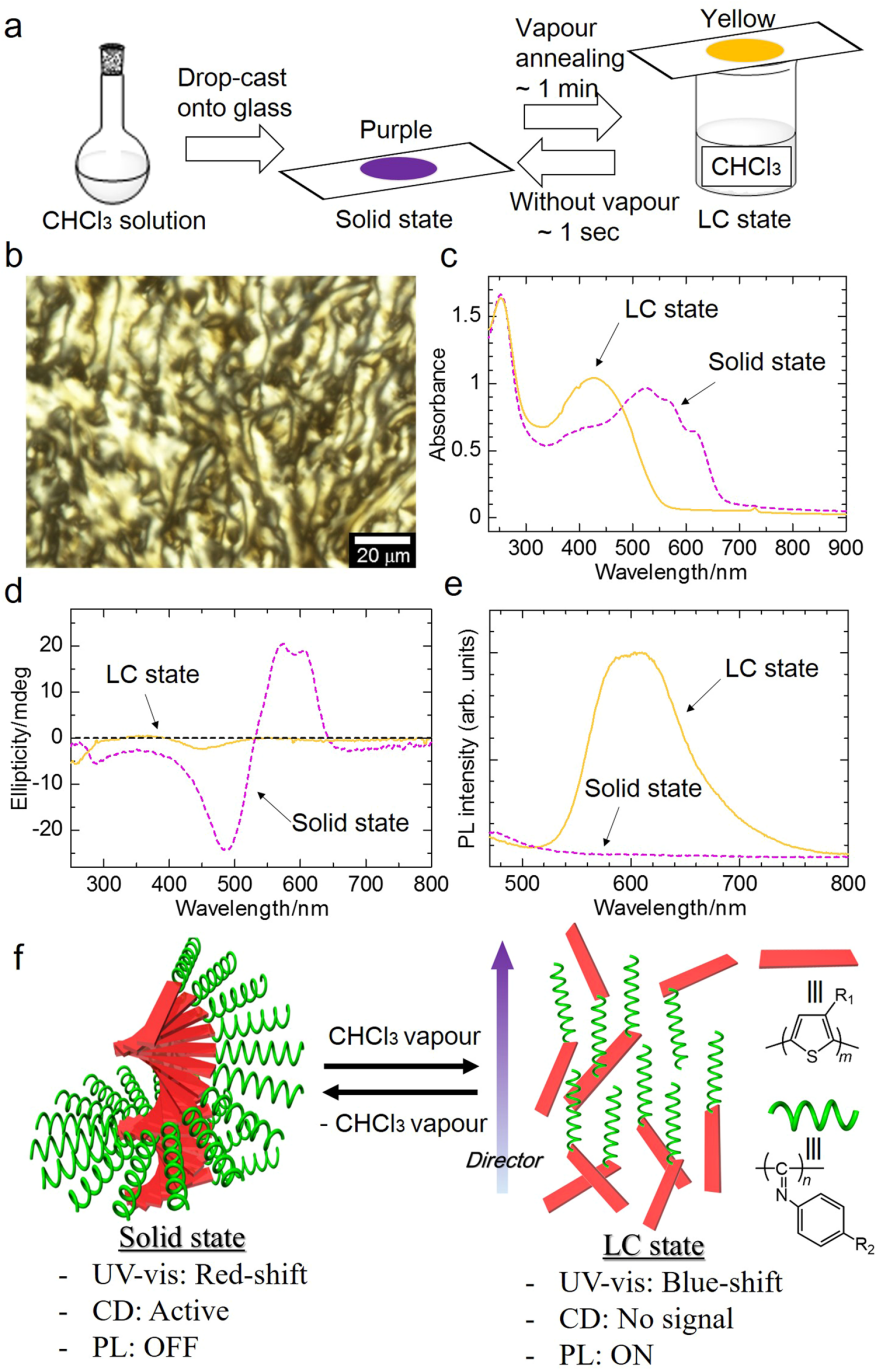
Vapour-induced liquid crystallinity of PTh-*b*-PPI. (**a**) Schematic representation of **PTh-** ***b*** **-PPI** film preparation and vapour exposure. The film shows purple in solid state and yellow in liquid crystal (LC) state (under chloroform vapour). (**b**) Polarising optical microscopy (POM) image of **PTh-** ***b*** **-PPI** film in LC state (when exposed to chloroform vapour). (**c**) Absorption, (**d**) CD and (**e**) PL spectra (λ_ex_ = 420 nm) of **PTh-** ***b*** **-PPI** film in solid state and LC state. (**f**) Schematic illustration of reversible phase-transition behaviour between solid state and nematic-like LC state of **PTh-** ***b*** **-PPI**. **PPI** blocks serve as mesogens and have orientation along the director.

We prepared **PTh-** ***b*** **-PPI** film by a drop-casting method from chloroform solution (2.0 mg/mL) onto a quartz substrate. Subsequently, **PTh-** ***b*** **-PPI** film was exposed to chloroform vapour annealing at room temperature. Very interestingly, the film colour changed from purple to transparent yellow in ~1 min in chroform vapour (Fig. 3a and Supplementary Video 1). More surprisingly, polarising optical microscopy (POM) observation evaluated the texture to be Schlieren structure of nematic liquid crystal phase (Fig. 3b,f). Other solvent vapours, such as tetrahydrofuran and dichloromethane, also induced the vapour-induced liquid crystal for the sample. After the removal of the vapour, the **PTh-** ***b*** **-PPI** film recovered their colour (yellow to purple) in ~1 sec. This reversible colour change is repeatable.

To examine the vapour-induced conformation change of **PTh-** ***b*** **-PPI** films, we performed optical measurements on the solid state (without vapour) and the LC state (with chloroform vapour). As shown in UV-vis spectra (Fig. 3c), **PTh-** ***b*** **-PPI** in solid state showed absorption peaks ~250 nm (π-π* transition of **PPI** block), 400 nm (n-π* transition of imine unit), and 527, 569, and 615 nm (**PTh** block). On the other hand, the LC-state **PTh-** ***b*** **-PPI** showed blue shift with the characteristic absorption peaks at ~250 nm (π-π* transition of **PPI** block) and ~427 nm (the sum of n-π* transition of imine unit and **PTh** block). The large blue shift was due to the reduced effective π-conjugated length, which is probably caused by permeation of the solvent vapour molecules intruded between main chains. In contrast, the absorption maximum of the **PPI** block at 250 nm did not change even exposed to the solvent vapour, implying that the **PPI** rigid helical coil was not subjected to intrusion by the vapour, and maintained a stable helical conformation. CD spectroscopy (Fig. 3d) shows **PTh-** ***b*** **-PPI** in solid state exhibited first-positive and second-negative Cotton effect, while the LC state of **PTh-** ***b*** **-PPI** showed no CD signals. The disappearance of CD spectra suggests the interruption of chiral interactions between PTh block by chloroform vapour. Additionally, PL spectroscopy (Fig. 3e) shows **PTh-** ***b*** **-PPI** in solid-state exhibited no signal in the visible region, while **PTh-** ***b*** **-PPI** in LC state showed photoluminescence at ~610 nm, which is ascribed to **PTh** block (excitation wavelength: 420 nm). The optical measurements suggest that the molecules in the vapour phase intrude between main chains of **PTh-** ***b*** **-PPI**, and depress the π-π interaction of **PTh** and expand the distance between the polymers, resulting in the blue-shift in UV-vis, the disappearance of the CD signal and the relaxation of the aggregation-induced quenching. It should be noted that **PTh-** ***b*** **-PPI** in LC state has blue-shifted absorption and red-shifted luminescence peaks compared to **PTh-** ***b*** **-PPI** in solution state (Fig. 2a,b). It implies that PTh chain in LC state is more twisted than in solution state, and the excited energy transfer between PTh chain may occur in the LC state

### Magnetic orientation of PTh-*b*-PPI with exposed to chloroform vapour

**Figure 4:**
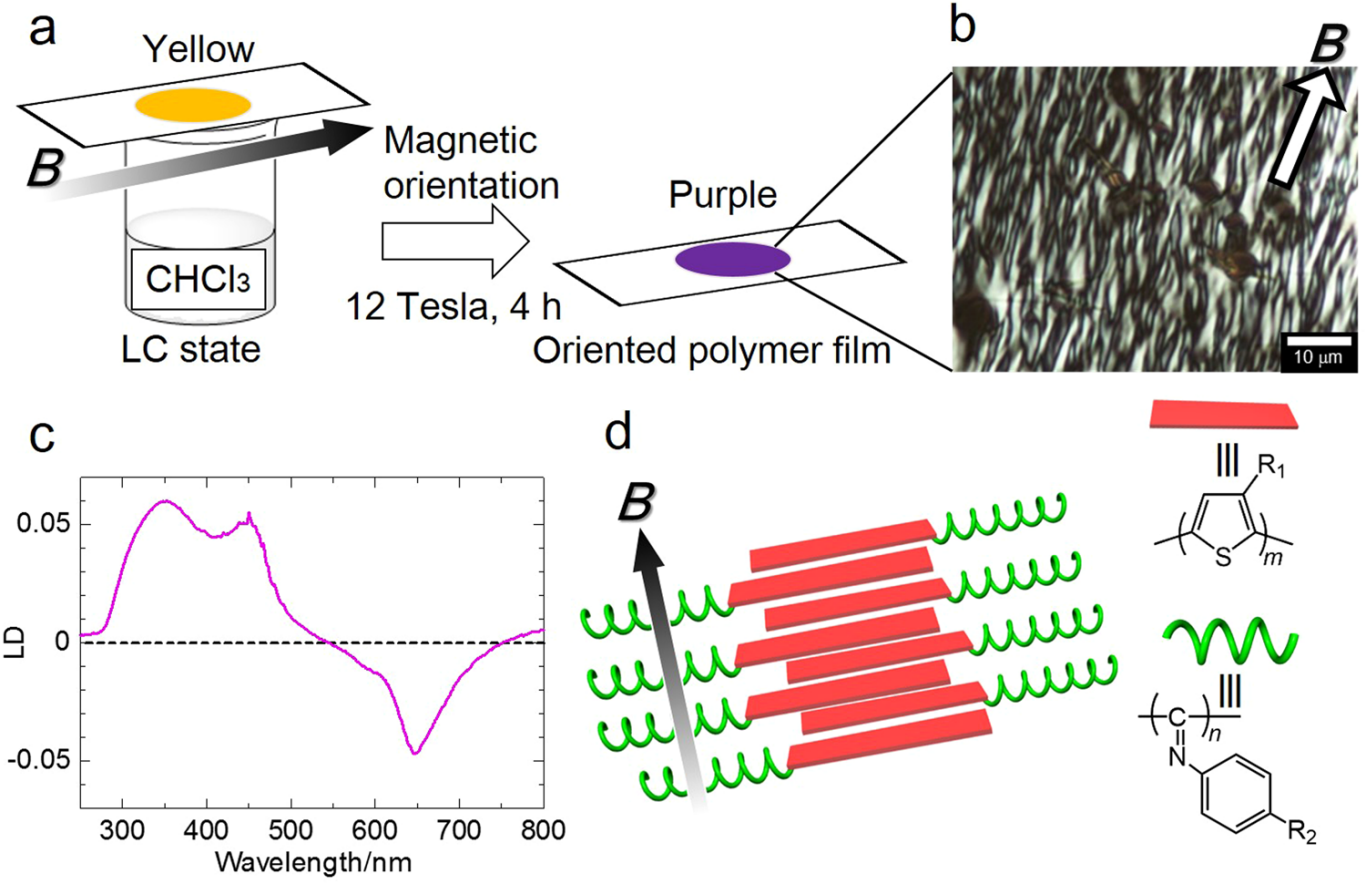
Magnetic orientation of PTh-*b*-PPI film. (**a**) Schematic representation of magnetic orientation of **PTh-** ***b*** **-PPI** film with exposure to chloroform vapour. (**b**) POM image of magnetically oriented **PTh-** ***b*** **-PPI** film. (**c**) Linear dichroism (LD) spectrum of magnetically oriented **PTh-** ***b*** **-PPI** film. (**d**) Schematic illustration of magnetically oriented **PTh-** ***b*** **-PPI**. Here, the parallel direction is along the direction of the magnetic field.

To demonstrate the merit of vapour-induced LC properties of **PTh-** ***b*** **-PPI** film, magnetic orientation of the film exposed to chloroform vapour was carried out (Fig. 4a). As shown in Fig. 4b, an intense magnetic field of 12 Tesla for 4 hours during the vapour-annealing successfully formed unidirectional orientation of **PTh-** ***b*** **-PPI** films (Fig. 4b). To investigate the unidirectionally oriented polymer structure, we employed linear dichroism (LD) spectroscopy. From the LD spectroscopy, we can determine the orientation of the sample,

$${\rm{LD}}={{\rm{OD}}}_{//}-{{\rm{OD}}}_{\perp }={\mathrm{log}}_{10}({{\rm{I}}}_{\perp }/{{\rm{I}}}_{//})$$

where, *OD* is optical density, *I* is intensity of transmitted light in parallel (//) and perpendicular (⊥) direction relative to the magnetic field. Figure 4c shows the negative signal (550–750 nm) derived from the **PTh** block, and positive peak (300–500 nm) coming from the **PPI** block. Generally, **PTh** (conjugated polymer) possesses π-π* electron transition moment along its backbone. That is, the positive signal indicates the **PTh** main chain is oriented perpendicular to the magnetic field. On the other hand, because the phenyl rings of the **PPI** helical block extend at the peripheral position of the helix core, the transition moment is perpendicular to the helical cylinder^28^. Therefore the negative signal means that the phenyl group in the side chain aligns parallel, and the helical axis perpendicular relative to the magnetic field (Fig. 4d). This alignment was realized by vapour-induced LC of **PTh-** ***b*** **-PPI** and the magnetically anisotropic susceptibility of **PPI** block.

### Discussion on vapour-induced liquid crystallinity of PTh-*b*-PPI

To investigate the origin of vapour-induced liquid crystallinity of **PTh-** ***b*** **-PPI**, homopolymers **PTh** and **PPI** were synthesised by the GRIM reaction and Ni-catalysed reaction, respectively. We prepared drop-cast film of **PTh** and **PPI** from the chloroform solution. Magnetic orientation of the homopolymers **PTh** and **PPI** under chloroform vapour was also carried out under chloroform vapour and the orientation was confirmed by the LD spectroscopy. Drop-cast **PTh** film showed no liquid crystal-like structure (Figure S4) and **PTh** chains could not be oriented by the magnetic field under chloroform vapour (Figures S5 and S6). This fact implies that **PTh** has no vapour-induced liquid crystallinity under chloroform vapour and thus does not have large domain size and/or moderate viscosity enough for susceptibility in alignment by magnetic field. On the other hand, drop-cast **PPI** film shows fan-shaped structure after chloroform vapour-annealing, indicating smectic-like super structure (Figure S4). Furthermore, **PPI** chains are macroscopically oriented by the magnetic field (Figures S5 and S6). This fact demonstrates **PPI** itself possesses intrinsically vapour-induced liquid crystallinity. Therefore, vapour-induced liquid crystallinity of **PTh-** ***b*** **-PPI** film is derived from intrinsically vapour-induced liquid crystallinity of PPI. We assume that the **PTh-** ***b*** **-PPI** film is very sensitive and it is swelled by the solvent vapour, resulting in low viscosity to form liquid crystal order. That is, the **PTh-** ***b*** **-PPI** film can form lyotropic LC state with slight amount of vapour molecules. Since lyotropic mesophase are seldom seen in CPs^29–31^, it is new strategy to introduce **PPI** block into conjugated backbone for realization of CPs with vapour-induced lyotropic LC.

### Self-recover mechanochromism of PTh-*b*-PPI

**Figure 5:**
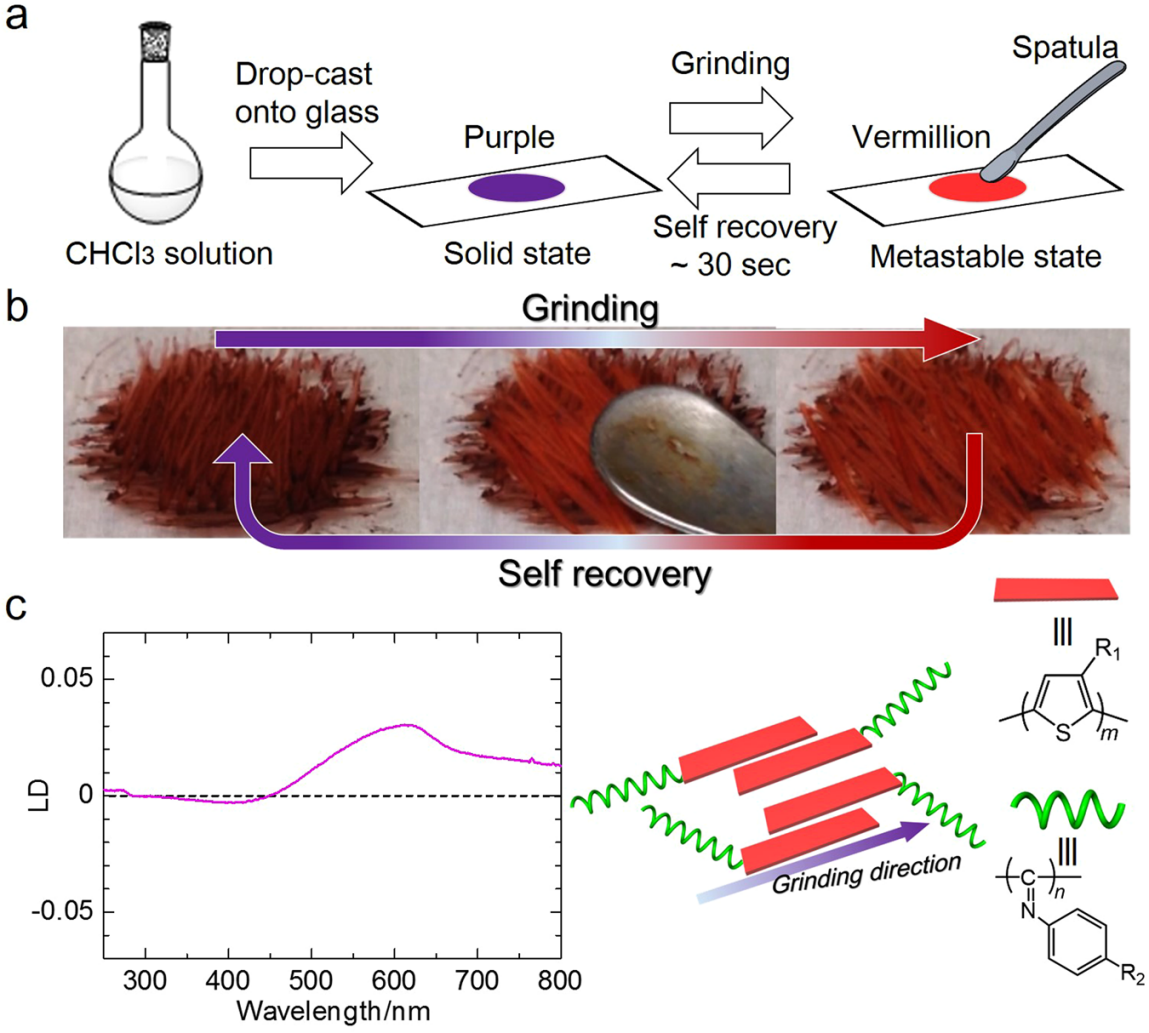
Self-recover mechanochromism of PTh-*b*-PPI. (**a**) Schematic representation of mechanical treatment of **PTh-** ***b*** **-PPI** films. (**b**) Photographs of **PTh-** ***b*** **-PPI** before and after mechanical stress. (**c**) LD spectrum of mechanically oriented **PTh-b-PPI** film and schematic illustration of mechanically oriented **PTh-** ***b*** **-PPI**. Here, the parallel direction is along the grinding direction.

Additionally, we performed a grinding test for the **PTh-** ***b*** **-PPI** solid film (Fig. 5a). Interestingly, we found that the film gradually turned to vermillion from purple during mechanical shearing. After the removal of the force, the colour recovered to purple under ambient conditions without any treatment in ~30 sec (Fig. 5b and Supplementary Video 2). In addition, the shear-stressed film showed anisotropic absorption between the parallel and perpendicular shear directions, which was confirmed by visible inspection through polarisers. As shown in the LD spectroscopy (Fig. 5c), the broad positive peak at 450–800 nm is ascribed to the **PTh** block. Thus the LD spectra indicates parallel orientation of the **PTh** chains to the shear direction. This implies that the rod shaped **PTh** block also can function as mesogen in shear-induced orientation. This shear-induced anisotropic orientation could be repeated, indicating a capability for overwriting/rewriting. This self-recovery mechanochromism is a unique phenomenon because many mechanochromism materials require aging treatment such as annealing, fuming and recrystallisation to regain original colour^32^. The colour change during the shear process probably comes from conformation and aggregation change of the **PTh** block. Although the detailed mechanism of the phenomenon is unclear, we assume that friction heat of shear stress causes conformation change of **PTh** block (Figure S7). After removal of the shear stress, the liquid crystalline **PPI** moiety probably facilitates the rearrangement to the original packing structure.

## Conclusions

We demonstrated vapour-induced liquid crystallinity and self-recover mechanochromisms of **PTh-** ***b*** **-PPI**. We found out new promising properties of rod-type helical coiled polymer **PPI** block: intrinsically vapour-induced liquid crystallinity and self-reassembling property. Introduction of PPI block offers moderate viscosity enough for **PTh-** ***b*** **-PPI** film to possess high sensitiveness to solvent vapour and self-recovery property against shear stress at ambient condition. In addition, PPI block in **PTh-** ***b*** **-PPI** produces the anisotropic magnetic susceptibility for magnetic alignment. As far as we know, **PTh-** ***b*** **-PPI** is the first material that exhibits vapour-induced liquid crystallinity. **PPI** helical structural motif has been employed mainly as chiral catalyst^33^, chiral separation^34^, chiral recognition^35^ because **PPI** can form one-handed helical conformation. Our study will pioneer new functionalities of rod-shaped helical polymers. Furthermore, magnetic orientation for vapour-exposed films provides a simple and convenient method to examine the films, offering a new orientation approach for block copolymers that possess anisotropic magnetic susceptibility units. We believe this block copolymer can be applied in phase-transition-type vapour sensors, pressure sensors, and as a memory medium for shear directions.

## Electronic supplementary material

Supplementary information

Video: Vapour annealing

Video: Grinding

## References

[1] Holtz JH, Asher SA (1997). Polymerized colloidal crystal hydrogel films as intelligent chemical sensing materials. Nature.

[2] Mura S, Nicolas J, Couvreur P (2013). Stimuli-responsive nanocarriers for drug delivery. Nat. Mater..

[3] Ahn SK, Kasi RM, Kim SC, Sharma N, Zhou Y (2008). Stimuli-responsive polymer gels. Soft Matter.

[4] Stuart MAC (2010). Emerging applications of stimuli-responsive polymer materials. Nat. Mater..

[5] Handbook of Liquid Crystals (Eds D. Demus, J.W. Goodby, G.W. Gray, H.-W. Spiess & V. Vill), Wiley-VCH,Weinheim, 1998.

[6] Schmitt V, Lequeux F, Pousse A, Roux D (1994). Flow behavior and shear induced transition near an isotropic/nematic transition in equilibrium polymers. Langmuir.

[7] Pujolle-Robic C, Noirez L (2001). Observation of shear-induced nematic-isotropic transition in side-chain liquid crystal polymers. Nature.

[8] Yoshio M, Mukai T, Ohno H, Kato T (2004). One-dimensional ion transport in self-organized columnar ionic liquids. J. Am. Chem. Soc..

[9] Ohtake T (2000). Liquid-crystalline complexes of mesogenic dimers containing oxyethylene moieties with LiCF3SO3: Self-organized ion conductive materials. Chem. Mater..

[10] Ichimura K (2000). Photoalignment of liquid-crystal systems. Chem. Rev..

[11] Tao Y, Zohar H, Olsen BD, Segalman RA (2007). Hierarchical nanostructure control in rod-coil block copolymers with magnetic fields. Nano Lett..

[12] Mei J, Bao Z (2013). Side chain engineering in solution processable conjugated polymers. Chem. Mater..

[13] Kawabata K, Saito M, Takemura T, Osaka I, Takimiya K (2017). Effects of branching position of alkyl side chains on ordering structure and charge transport property in thienothiophenedione and quinacridone-based semiconducting polymers. Polymer Journal.

[14] Berggren M (1994). Green Electroluminescence in Poly(3-cyclohexylthiophene) light emitting diodes. Adv. Mater..

[15] Marsella MJ, Newland RJ, Carroll PJ, Swager TM (1995). Ionoresistivity as a highly sensitive sensory probe: investigations of polythiophenes functionalized with calix [4] arene-based ion receptors. J. Am. Chem. Soc..

[16] Langeveld-Voss BMW, Janssen RAJ, Meijer EW (2000). On the origin of optical activity in polythiophenes. J. Mol. Struct..

[17] Onouchi H (2008). Two-and three-dimensional smectic ordering of single-handed helical polymers. J. Am. Chem. Soc..

[18] Dama M, Berger S (2012). Polyisocyanides as a new alignment medium to measure residual dipolar couplings for small organic molecules. Org. Lett..

[19] Wu ZQ, Ono RJ, Chen Z, Bielawski CW (2010). Synthesis of poly (3-alkylthiophene)-block-poly (arylisocyanide): Two sequential, mechanistically distinct polymerizations using a single catalyst. J. Am. Chem. Soc..

[20] Wu Z-Q (2013). One‐pot synthesis of conjugated poly (3-hexylthiophene)-b-poly (phenyl isocyanide) hybrid rod–rod block copolymers and its self‐assembling properties. Polym. Sci. A. Polym. Chem..

[21] Wu ZQ (2012). Synthesis of conjugated diblock copolymers: two mechanistically distinct, sequential living polymerizations using a single catalyst. Polym. Chem..

[22] Liu N (2013). Solvent-Induced White-Light Emission of Amphiphilic Rod–Rod Poly (3-triethylene glycol thiophene)-block-poly (phenyl isocyanide) Copolymer. Macromolecules.

[23] Zhu Y-Y (2014). Poly (3-hexylthiophene)-block-poly (5,8-di-p-tolylquinoxaline-2,3-diyl) conjugated rod-rod copolymers: one pot synthesis, self-assembly and highly selective sensing of cobalt. RSC Adv..

[24] Loewe RS, Ewbank PC, Liu J, Zhai L, McCullough RD (2001). Regioregular, head-to-tail coupled poly (3-alkylthiophenes) made easy by the GRIM method: investigation of the reaction and the origin of regioselectivity. Macromolecules.

[25] Miyakoshi R, Yokoyama A, Yokozawa T (2005). Catalyst-transfer polycondensation. Mechanism of Ni-catalyzed chain-growth polymerization leading to well-defined poly(3-hexylthiophene). J. Am. Chem. Soc..

[26] Vandeleene S (2011). Influence of the Supramolecular Organization on the Magnetic Properties of Poly (3-alkylthiophene)s in Their Neutral State. Macromolecules.

[27] Bidan G, Guillerez S, Sorokin V (1996). Chirality in regioregular and soluble polythiophene: an internal probe of conformational changes induced by minute solvation variation. Adv. Mater..

[28] Feng F, Miyashita T, Takei F, Onitsuka K, Takahashi S (2001). Formation of an Optically Active Helical Polyisocyanide Langmuir-Blodgett Film. Chem. Lett..

[29] Kim BG (2013). A molecular design principle of lyotropic liquid-crystalline conjugated polymers with directed alignment capability for plastic electronics. Nat. Mater..

[30] Bridges CR, Ford MJ, Popere BC, Bazan GC, Segalman RA (2016). Formation and Structure of Lyotropic Liquid Crystalline Mesophases in Donor–Acceptor Semiconducting Polymers. Macromolecules.

[31] Goto H, Wang A, Nimori S, Kawabata K (2013). Mechanical orientation in thermotropic liquid crystal state and magnetic orientation in solvent evaporation process via lyotropic liquid crystal state of an amphotropic low-bandgap liquid-crystalline π-conjugated polymer. Liquid Crystals.

[32] Sagara Y, Kato T (2009). Mechanically induced luminescence changes in molecular assemblies. Nat. Chem..

[33] Miyabe T, Hase Y, Iida H, Maeda K, Yashima E (2009). Synthesis of functional poly(phenyl isocyanide) s with macromolecular helicity memory and their use as asymmetric organocatalysts. Chirality.

[34] Miyabe T, Iida H, Ohnishi A, Yashima E (2012). Enantioseparation on poly(phenyl isocyanide)s with macromolecular helicity memory as chiral stationary phases for HPLC. Chem. Sci..

[35] Ishikawa M, Maeda K, Mitsutsuji Y, Yashima E (2004). An unprecedented memory of macromolecular helicity induced in an achiral polyisocyanide in water. J. Am. Chem. Soc..

