# Supplementary material for: Vapour-Induced Liquid Crystallinity and Self-Recovery Mechanochromism of Helical Block Copolymer
Source: Sci Rep. 2017 Jun 21;7:3948. doi: 10.1038/s41598-017-03948-7 (PMC5479860; doi:10.1038/s41598-017-03948-7)
Supplement: Supplementary file 1 — Supplementary information [file 41598_2017_3948_MOESM1_ESM.docx]

Supplementary Information for:

Vapour-Induced Liquid Crystallinity and Self-Recovery Mechanochromism of Helical Block Copolymer

Hiroki Hayashi^[a]^, Tomokazu Iseki^[a]^, Shigeki Nimori^[b]^, Hiromasa Goto*^[a]^

[a] Graduate school of Pure and Applied Sciences, Institute of Materials Science

University of Tsukuba, Tsukuba, Ibaraki 305-8573, Japan

E-mail: gotoh@ims.tsukuba.ac.jp

[b] Tsukuba Magnet Laboratory National Institute for Materials Science (NIMS) 3-13 Sakura, Tsukuba, Ibaraki 305-0003, Japan

Table of Contents-

**General Remarks**

**Materials**

**Method for optical measurement under vapour condition**

**Chart S1**

**Synthetic Procedures**

**Synthesis of Br-terminated chiral alkyl chain**

**Synthesis of Thiophene monomer**

**Synthesis of Phenylisocyanide monomer**

**Synthesis of PTh-*b*-PPI copolymer**

**Synthesis of PTh homopolymer**

**Synthesis of PPI homopolymer**

**Spectroscopic and Polarizing Optical Microscopy (POM) data**

**Figure S1**. NMR spectrum of PTh-*b*-PPI.

**Figure S2**. IR spectrum of PTh-*b*-PPI.

**Figure S3**. In-situ absorption and CD spectra changes of PTh-b-PPI film under iodine vapour.

**Figure S4**. POM images of PTh-*b*-PPI, PTh and PPI in solid state at different magnification.

**Figure S5**. POM images of PTh-*b*-PPI, PTh and PPI in solid state after magnetic orientation with exposed chloroform vapour at different magnification.

**Figure S6**. LD spectra of magnetically treated PTh-*b*-PPI, PTh and PPI.

**Figure S7**. POM images of themochromism behaviour of PTh-*b*-PPI in solid on glass substrate.

**Figure S8**. UV-vis absorption spectra of PTh-*b*-PPI in tetrahydrofuran (THF), dichloromethane, chloroform and tetrachloromethane (0.02 mg/ml). The polymer is insoluble in acetonitrile, 1,4-dioxane, *N,N*-dimethylformamide (DMF) and dimethyl sulfoxide (DMSO).

**Figure S9**. CIE colour space of PTh-*b*-PPI solution in tetrahydrofuran (THF), dichloromethane, chloroform and tetrachloromethane (concentration: 0.02 mg/ml).

**Figure S10**. POM images of poly(decyl-phenylisocyanide) films on glass substrate at different magnification.

**References**

**General Remarks.** ^1^H NMR and ^13^C NMR spectra were recorded using a JNM-ECS (JEOL, 400 MHz). Chemical shifts were given in parts per million and coupling constant (J) in Hz. Microscopic observations were performed under crossed Nicols using a Nikon Eclipse LV100 polarizing optical microscope (POM) equipped with a JHT TH-600PM and a L-600 heating and cooling stage (Linkam). UV-vis absorption spectra were obtained using a JASCO V-630 UV-vis spectrophotometer. Circular dichroism (CD) and linear dichroism (LD) were obtained using a JASCO J-720 spectrometer. The PL spectra were collected employing F-4500 spectrometer (Hitachi). The molecular weights of the polymers were evaluated with gel permeation chromatography (GPC) relative to polystyrene standard (eluent: THF).IR spectra were recorded using Jasco FT-IR 500 spectrometer using KBr method. Differential scanning calorimetry (DSC) was performed using a Seiko Instrument Exstar7000 DSC under a nitrogen atmosphere at a constant heating and cooling rate of 5 °C min^−1^. Magnetic orientation was carried out with a drum type cryogenfree superconducting magnet (Japan Magnet Technology, JMT).

**Materials.** The commercially available reagents were received from Nacalai Tesque Co., Ltd., Aldrich, Kanto Chemical Co. and TCI chemicals and used without further purification. The common organic solvents such as dichloromethane and tetrahydrofuran (THF) were distilled and handled in a moisture-free atmosphere. (3*S*)-1-Bromo-3,7-dimethyloctan and the monomers, 6 and 11 were prepared according to the literature procedures.^[1,2,3]^ The polymers, PTh-*b*-PPI, PTh, PPI and poly(dodecylphenylisocyanide) ^[3]^ were also prepared by the previously reported method.^[3]^

**Method for optical measurement under vapour condition.** Optical measurements under chloroform or iodine vapours were conducted using the setup as shown in Chart 1. First, chloroform (0.3 mL) or iodine crystal (12 mg) were put into a quartz cell. Then, a plastic stand was placed in the cell to prevent the direct contact between the polymer film and chloroform liquid or iodine crystal. Finally, drop-cast film on the quartz substrate was set on the stand and then, the lid was sealed with Parafilm.


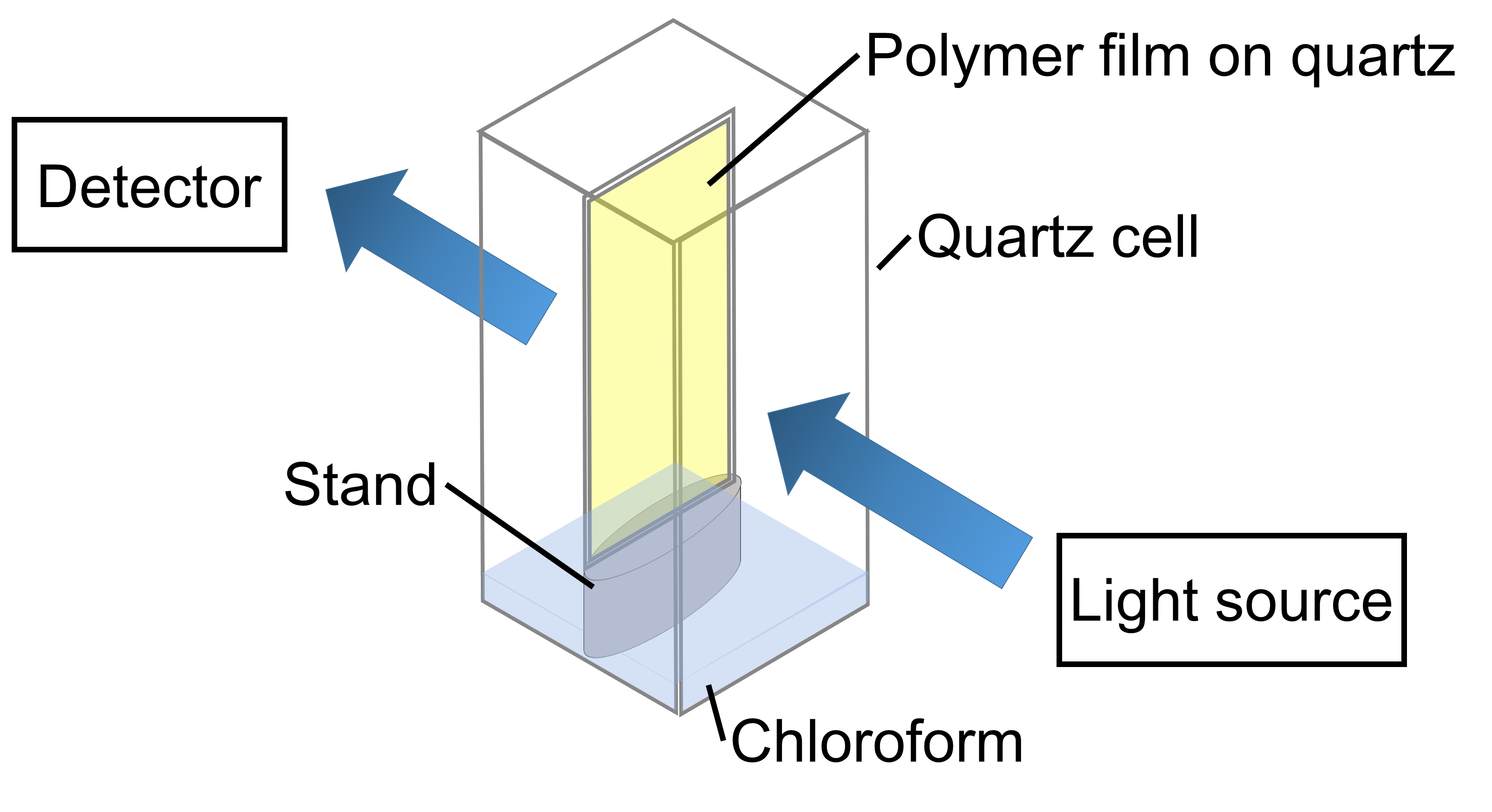


Chart S1. Illustration of optical measurement under vapours.

**Synthesis of Br-terminated chiral alkyl chain**

**(*S*)-3,7-Dimethyl-1-octanol (2)** A mixture of compound 2 (3 g, 19.2 mmol) and Pd/C (0.152 g, 5% by weight) in ethyl acetate was stirred at room temperature under H_2_ atmosphere. After 2 days stirring, the crude product was filtered with Celite to yield colourless liquid (2.85 g, 17.98 mmol, 93.6%). ^1^H NMR (400 MHz, CDCl_3_): δ 3.71-3.65 (m, 2H), 1.61-1.50 (m, 3H), 1.40-1.26 (m, 5H), 1.17-1.11 (m, 2H), 0.90-0.85 (m, 9H). ^13^C NMR (100 MHz, CDCl_3_): δ 61.27, 39.99, 39.26, 37.37, 29.50, 27.97, 24.68, 22.70, 22.60, 19.65.

**(3*S*)-1-Bromo-3,7-dimethyloctan (3)** To a solution of compound 3 (2.8 g, 17.7 mmol) and triphenylphosphine (5.13 g, 19.5 mmol) in dichloromethane (30 mL) was added NBS (3.32 g, 18.6 mmol) in portions. After stirring for 4 h at room temperature, the reaction mixture was evaporated and extracted with *n*-hexane/H_2_O followed by MgSO_4_ drying. The crude product was purified by column chromatography (eluent: *n*-hexane) to yield colourless oil (2.49 g, 11.2 mmol, 63%). ^1^H NMR (400 MHz, CDCl_3_): δ 3.50-3.37 (m, 2H), 1.92-1.84 (m, 1H), 1.71-1.64 (m, 2H), 1.15-1.47 (m, 1H), 1.30-1.26 (m, 3H), 1.19-1.09 (m, 3H), 0.89-0.86 (m, 9H). ^13^C NMR (100 MHz, CDCl_3_): δ 77.32, 77.00, 76.68, 40.06, 39.15, 36.25, 32.25, 31.65, 27.93, 24.53, 22.68, 22.57, 18.94.

**Synthesis of Thiophene monomer.**

**3-((3*S*)-3,7-Dimethyl-octyl)-thiophene (5)** To a suspension of magnesium turnings (0.380 g, 15.6 mmol) in THF (10 mL) was added iodine (0.15 g). The suspension was refluxed for 2 h to activate magnesium resulting in yellow solution. **5** (3.18 g, 14.4 mmol) was added to the yellow mixture at 0°C and then refluxed for another 1h. The resulting gray solution was cooled to room temperature. To a another flask were added **6** (1.01 mL, 10. 66 mmol) and Ni(dppp)Cl_2_ (0.07 g, 0.13 mmol) and dissolved in THF (10 mL). To this solution was added the gray solution dropwise at 0°C and then refluxed for 21h. The reaction was quenched by 2N hydrochloric acid at room temperature. The reaction mixture was evaporated and extracted with diethyl ether/H_2_O followed by MgSO_4_ drying. The crude product was purified by column chromatography (eluent: *n*-hexane) to yield the target product (1.76 g, 7.85 mmol, 74%). ^1^H NMR (400 MHz, CDCl_3_): δ 7.25-7.22 (m, 1H), 6.95-6.91 (m, 2H), 2.65-2.58 (m, 2H), 1.67-1.62 (m, 1H), 1.53-1.42 (m, 4H), 1.32-1.10 (m, 6H), 0.91-0.87 (m, 10H). ^13^C NMR (100 MHz, CDCl_3_): δ 143.44, 128.27, 125.04, 119.60, 39.30, 37.80, 37.12, 32.46, 27.96, 27.84, 24.69, 22.70, 22.61, 19.56.

**2,5-Dibromo-3-((3*S*)-3,7-dimethyl-octyl)-thiophene (6)** To a solution of **7** (1.76 g, 7.9 mmol) in DMF (55 mL) was added NBS (3.07 g, 17.3 mmol) in portions at 0°C. After stirring for 2 days at room temperature under N_2_ atmosphere, the reaction was quenched by saturated NaHCO_3_ aq. The reaction mixture was extracted with EtOAc/H2O followed by MgSO_4_ drying. The crude product was purified by column chromatography (eluent: *n*-hexane) to yield **1b** (2.75 g, 7.2 mmol, 92%). ^1^H NMR (400 MHz, CDCl_3_): δ 6.78 (s, 1H), 2.54-2.47 (m, 2H), 1.56-1.10 (m, 11H), 0.92-0.90 (d, 3H), 0.87-0.86 (d, 6H). ^13^C NMR (100 MHz, CDCl_3_): δ 143.15, 130.89, 110.31, 107.74, 39.29, 36.95, 36.71, 32.87, 27.98, 27.17, 24.65, 22.72, 22.64, 19.55.

**Synthesis of Phenylisocyanide monomer**

**Octyl 4-nitrobenzoate (8).** To a solution of nitrobenzoic acid **7** (2.99 g, 17.9 mmol) and 1-bromooctane (4.67 mL, 26.84 mmol) in 2-butanone (100 mL) was added potassium carbonate (3.96 g, 28.6 mmol) and then refluxed for 16 h. After reaction, the reaction solution was evaporated and extracted with chloroform/H_2_O followed by MgSO_4_ drying. The residue was purified by column chromatography (eluent: *n*-hexane, chloroform) to yield **9** (3.62 g, 12.9 mmol, 72.5%). ^1^H NMR (400 MHz, CDCl_3_): δ 8.29 (d, 2H, *J* = 8.8 Hz), 8.21 (d, 2H, *J* = 8.7 Hz), 4.37 (t, 2H, *J* = 6.6 Hz), 1.81-1.76 (m, 2H), 1.46-1.29 (m, 10H), 0.89 (t, 3H, *J* = 5.3 Hz). ^13^C NMR (100 MHz, CDCl_3_): δ 164.75, 150.55, 135.90, 130.64, 123.51, 66.12, 31.76, 29.19, 29.16, 28.58, 25.96, 22.61, 14.07.

**Octyl 4-aminobenzoate (9)** Compound 5 was synthesized as the same procedure as compound 3. Yield: **10** (2.59 g, 80.6%). ^1^H NMR (400 MHz, CDCl_3_): δ 7.87-7.83 (m, 2H), 6.65-6.62 (m, 2H), 4.25 (t, 2H, *J* = 6.6 Hz), 4.04 (s, 2H), 2.88-1.27 (m, 12H), 0.88 (t, 3H, *J* = 6.9 Hz). ^13^C NMR (100 MHz, CDCl_3_): δ 166.74, 150.66, 131.53, 120.15, 113.75, 64.52, 31.79, 29.26, 29.19, 28.80, 26.05, 22.62, 20.39, 14.08.

**Octyl 4-formamidobenzoate (10).** A solution of formic acid (0.90 mL, 23.8 mmol) and acetic anhydride (2.25 mL, 23.8 mmol) was stirred at 54°C and then another solution of 9 (2.7 g, 10.8 mmol) in diethylether (50 mL) was added dropwise at 0°C. The mixture was stirred for 28 h at room temperature and then quenched by 10% aq NaHCO_3_ solution. The crude product was extracted with EtOAc/H_2_O and purified by column chromatography (eluent: chloroform/ethylacetate = 4/1) to yield white solid (2.50 g, 83.1%). **^1^**H NMR (400 MHz, CDCl_3_): δ 8.83 (d, 0.44H, NH in trans, *J* = 11 Hz), 8.44 (d, 0.56H, NH in cis, *J* = 1.4 Hz), 8.27 (d, 0.44H, HCO in trans, *J* = 11 Hz), 8.06-8.02 (m, 2H, Hm to NH in cis and Hm to NH in trans), 7.64 (d, 1.2H, Ho to NH in cis, *J* = 9.2 Hz), 7.26 (s, 0.56H, HCO in cis), 7.14 (d, 0.88H, Ho to NH in trans, *J* = 8.2 Hz), 4.33-4.28 (m, 4H), 1.79-1.28 (m, 12H), 0.88 (t, 3H, *J* = 6.9 Hz). ^13^C NMR (100 MHz, CDCl_3_): δ 166.03, 165.82, 161.73, 158.89, 140.79, 131.48, 130.83, 127.03, 126.54, 119.01, 117.15, 65.30, 65.17, 31.77, 29.23, 29.18, 28.69, 26.03, 22.63, 14.09.

**Octyl 4-isocyanobenzoate (11)**. To an oven-dried Schlenk flask were added compound 10 (277 mg, 1.0 mmol), dichloromethane (8 mL) and triethylamine (0.38 mL, 2.7 mmol) under Ar atmosphere. POCl_3_ (0.10 mL, 1.1 mmol) was added dropwise slowly at 0°C and then stirred at 0°C for 10 min. The reaction mixture was warmed up to room temperature and stirred for another 1 h. Then Na_2_CO_3_aq (10%) 20 mL was added and extracted with chloroform followed by MgSO_4_ drying. The residue was purified by column chromatography (eluent: hexane/ CH_2_Cl_2_ = 7/3) to yield phenylisocyanide monomer (244 mg, 94%). IR (KBr, cm^–1^): 2122 (ν_C≡N_), 1724 (ν_C=O_ ester).　 **^1^**H NMR (400 MHz, CDCl_3_): δ 8.08 (d, 2H, *J* = 8.8 Hz), 7.45 (d, 2H, *J* = 8.0 Hz), 4.33 (t, 2H, *J* = 6.8 Hz), 1.77 (quin, 2H, *J* = 6.8 Hz), 1.47 – 1.28 (m, 10H). 0.89 (t, 3H, *J* = 6.8 Hz). ^13^C NMR (100 MHz, CDCl_3_): δ 165.04, 131.31, 130.78, 126.40, 65.75, 37.74, 29.18, 29.14, 28.59, 25.96, 22.60, 14.05.

**Synthesis of PTh-*b*-PPI copolymer**

**PTh-*b*-PPI copolymer.** To an oven-dried Schlenk flask were added thiophene monomer (191 mg) and dissolved in dry THF (5 mL) under Ar atmosphere. A THF solution of *i*-PrMgBr⋅LiCl (1 mol/L, 0.5 mL) was added dropwise over 30 min under Ar atmosphere and the reaction mixture was stirred at room temperature for 90 min. Then Ni(dppp)Cl_2_ (2.7 mg) was added at room temperature. The colour of reaction mixture turned to red. The growth of molecular weight was monitored by GPC. After 1h stirring, the growth of molecular weight was ceased to increase (Mn = 6000, PDI = 7.03). Subsequently, to the same flask was added isocyanide monomer (130 mg) and stirred for 1 h at room temperature. The colour of reaction mixture turned to black. Then the reaction mixture was poured into excess methanol and collected by filtration followed by vacuum drying to yield the PTh-*b*-PPI (209 mg, 65%). The GPC result for PTh-*b*-PPI: *Mn* = 16000, *PDI* = 4.47.

**Synthesis of PTh homopolymer**

**PTh homopolymer**. To an oven-dried Schlenk flask were added thiophene monomer **6** (96 mg) and dissolved in dry THF (2 mL) under Ar atmosphere. A THF solution of i-PrMgBr⋅LiCl (1 mol/L, 0.26 mL) was added dropwise over 30 min under Ar atmosphere and the reaction mixture was stirred at room temperature for 90 min. Then Ni(dppp)Cl_2_ (1.4 mg) was added at 0°C. The reaction mixture was stirred overnight at room temperature. Then the reaction mixture was poured into excess methanol and collected by centrifugation followed by vacuum drying to yield the PT.

**Synthesis of PPI homopolymer**

**PPI homopolymer**. To an oven-dried Schlenk flask was added phenylisocyanide monomer **11** (206 mg mg) dissolved in dry dichloromethane (5 mL) and NiCl_2_ (7.1 mg) under Ar atmosphere. The reaction mixture was stirred at room temperature for 23 h. Then the mixture was poured into excess methanol and acetone followed by vacuum drying to yield PPI homopolymer.


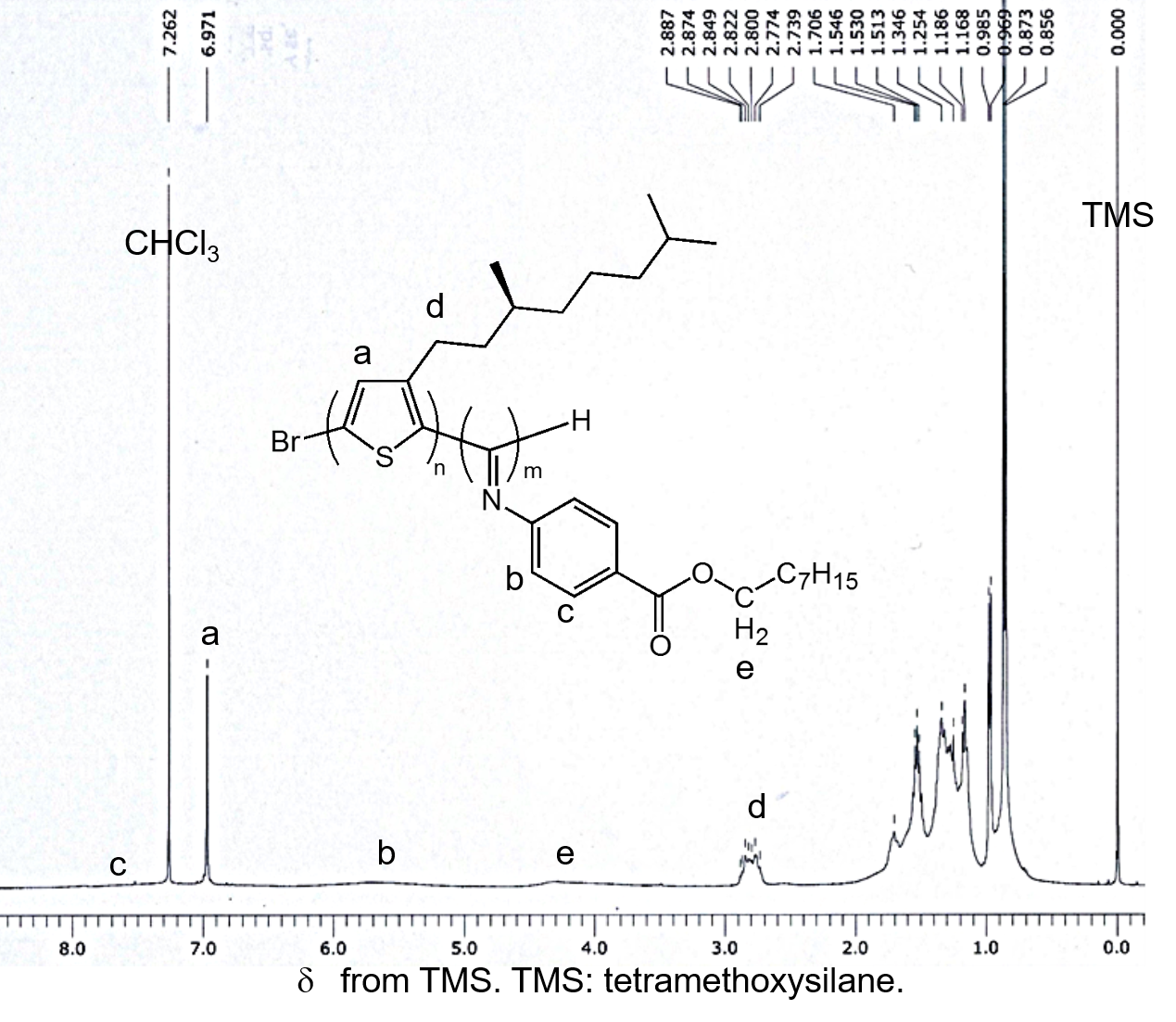


**Figure S1**. NMR spectrum of PTh-*b*-PPI.


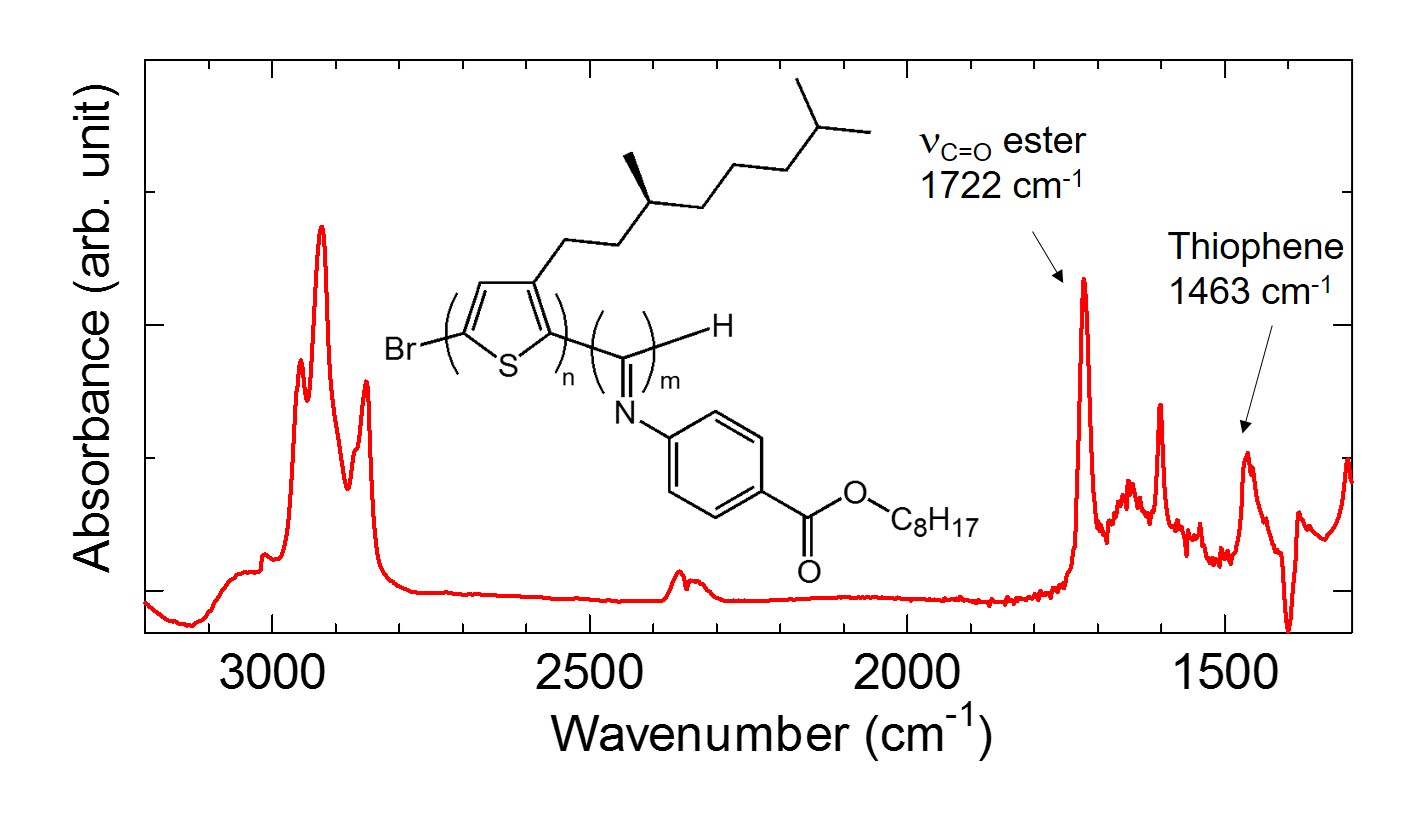


**Figure S2**. Infrared (IR) absorption spectrum of PTh-*b*-PPI.

PT-*b*-PPI was characterised by NMR and IR spectroscopy. In NMR spectrum (Figure S1), the peaks derived from PTh block were clearly observed. The sharp peak at 6.97 ppm is coming from the β position hydrogen of thiophene and this non-split peak indicates high regioregularity of PTh. The broad peak at around 5.7 ppm is attributed to hydrogens bonded to PPI phenyl ring and the broad peak at around 4.3 ppm is originated from CH_2_ bonded to ester. In IR spectrum, the very sharp PPI ester peak was observed at 1722 cm^-1^. The PTh peak was also seen at 1463 cm^-1^.


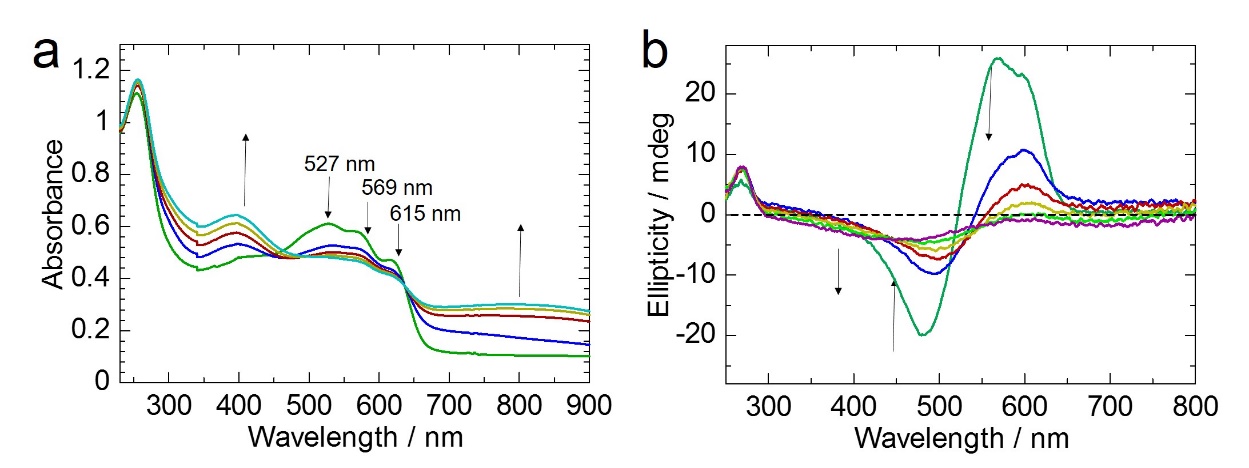


**Figure S3**. In-situ absorption and circular dichroism absorption (CD) spectra changes of PTh-b-PPI film under iodine vapour.

The degree of oxidation gives large conformation changes to **PTh** (π-conjugated polymers). We carried out the chemical doping with iodine vapour for the solid state **PTh-*b*-PPI** film (Figure S3). Up to date, many researchers investigated polymer chiral conformations by the comparison between neutral and oxidation state of the polymer using oxidation/reduction reagents^[4]^ or use of electrochemistry methods.^[5]^ The iodine vapour exposure to the **PTh-*b*-PPI** film resulted in the oxidation and the formation of polaron (radical cation) state. The spectra ranging in 700-900 nm increased as the doping time (0 - 9 min), which is typical of common polaron state of **PTh** (Figure S3a). On the contrary, the absorption peaks at 527, 569, 615 nm became smaller. In CD spectra (Figure S3b), the signal originating from the **PTh** right-handed aggregation became smaller and blueshifted to at around 450 nm. However, even after heavy doping by iodine, the **PTh-*b*-PPI** film still showed the CD signal. This indicates that undoped chromophores with shorter conjugation length remain and the polymer chains maintain the chiral orientation in the doped state.^[6]^

**PPI** moiety also generated a radical cation state by the doping of iodine vapour. The generation of radical cations at N atoms in the **PPI** molecular chain is consistent with an iminyl radical, which was confirmed by the electron spin resonance (ESR) measurement.^[7]^


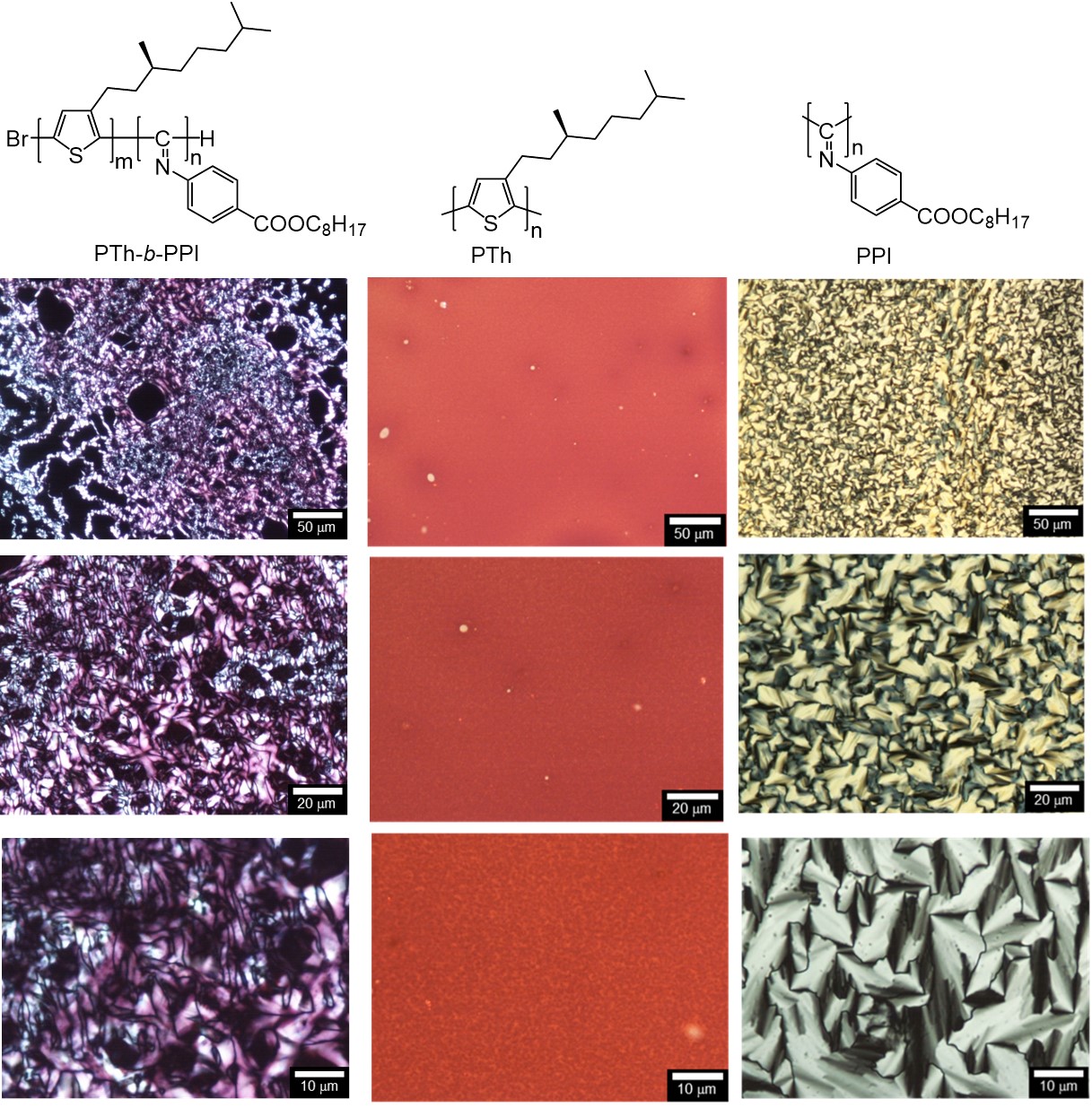


**Figure S4.** Polarizing optical microscopy (POM) images of PTh-*b*-PPI, PTh and PPI films in solid state at different magnification.


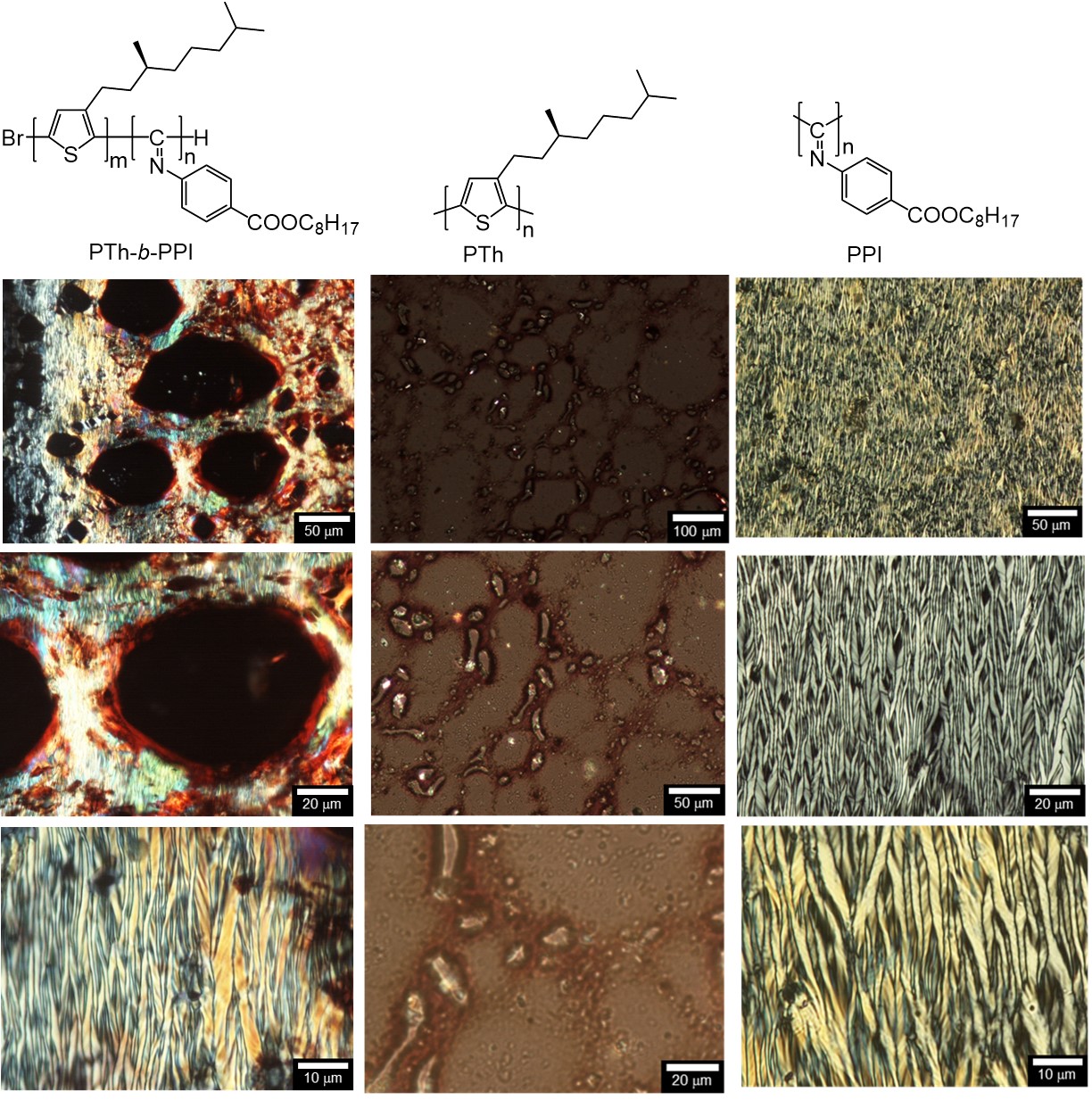


**Figure S5**. POM images of PTh-*b*-PPI, PTh and PPI films in solid state after magnetic orientation with exposed chloroform vapour at different magnification.


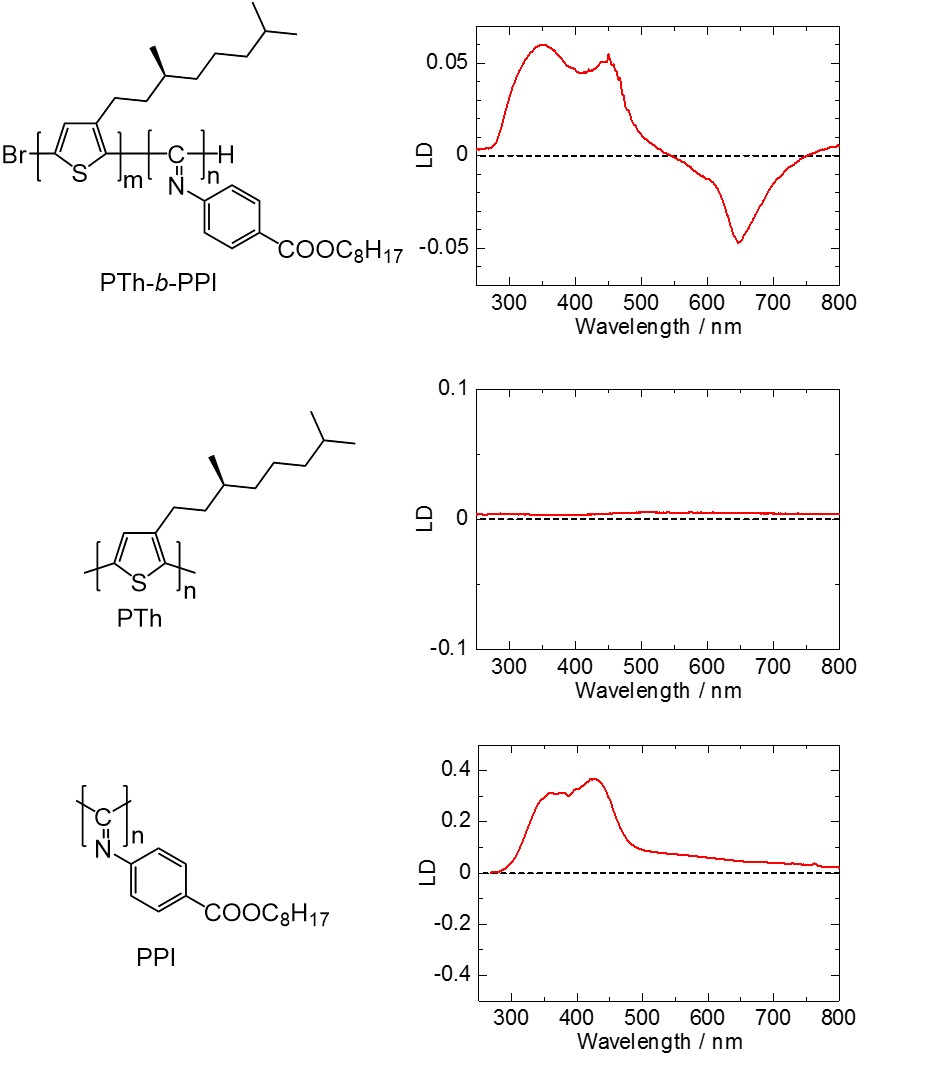


**Figure S6.** Linear dichroism (LD) spectra of magnetically treated PTh-*b*-PPI, PTh and PPI solid films.


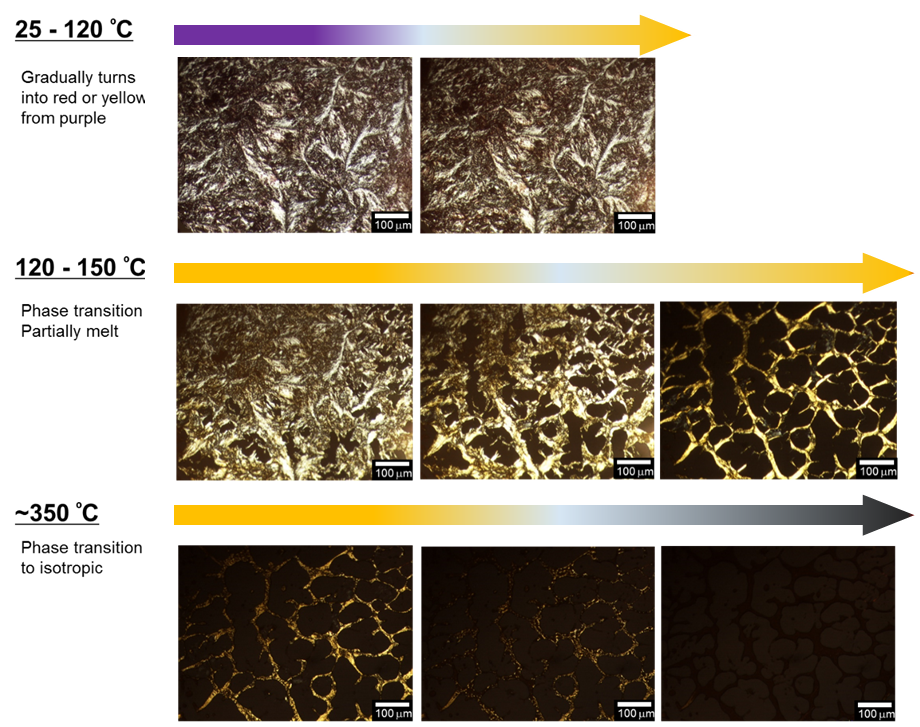


**Figure S7**. POM images of themochromism behaviour of PTh-*b*-PPI film on glass substrate.

To investigate the thermo-properties of PT-b-PPI, the PT-b-PPI film was monitored using polarizing optical microscopy (POM) on the temperature variable stage. The temperature range was 25 – 350°C and heating rate was 10°C/min. In the range of 25 – 120°C, the colour of the film turned to yellow from purple. This state was liquid crytal phase. On the heating process in 120 - 150°C, phase transition occurred and the film formed network structure. This partial melting was originated from the glass transition of PTh block. In the range of 150 – 350°C, the film showed no changes. Finally at 350°C, the PT-b-PPI was completely melt, indicating the phase transition to isotropic phase.


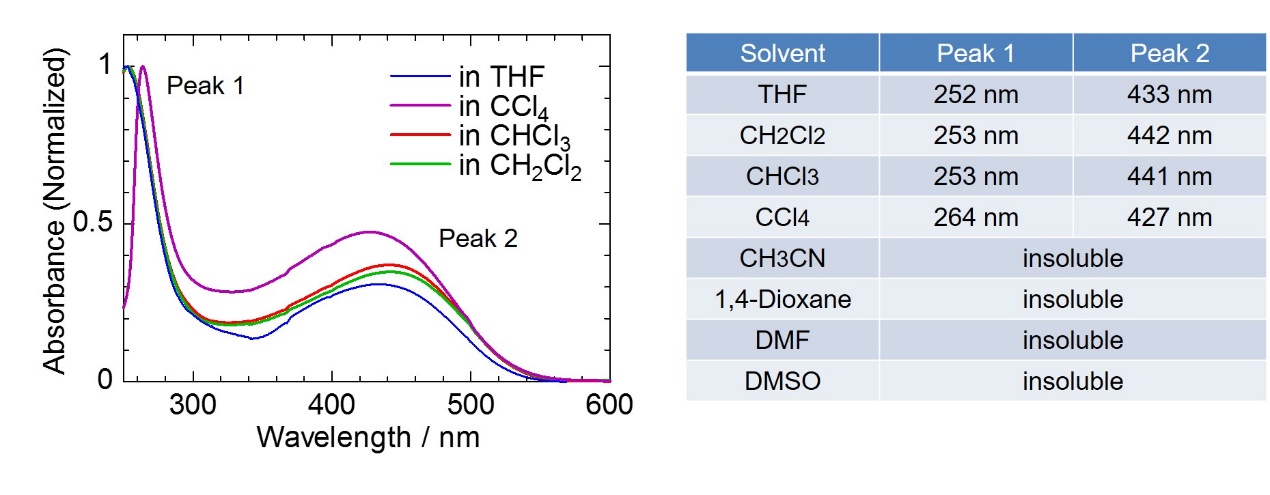


**Figure S8**. UV-vis absorption spectra of PTh-*b*-PPI in tetrahydrofuran (THF), dichloromethane, chloroform and tetrachloromethane (0.02 mg/ml). The polymer is insoluble in acetonitrile, 1,4-dioxane, *N,N*-dimethylformamide (DMF) and dimethyl sulfoxide (DMSO).


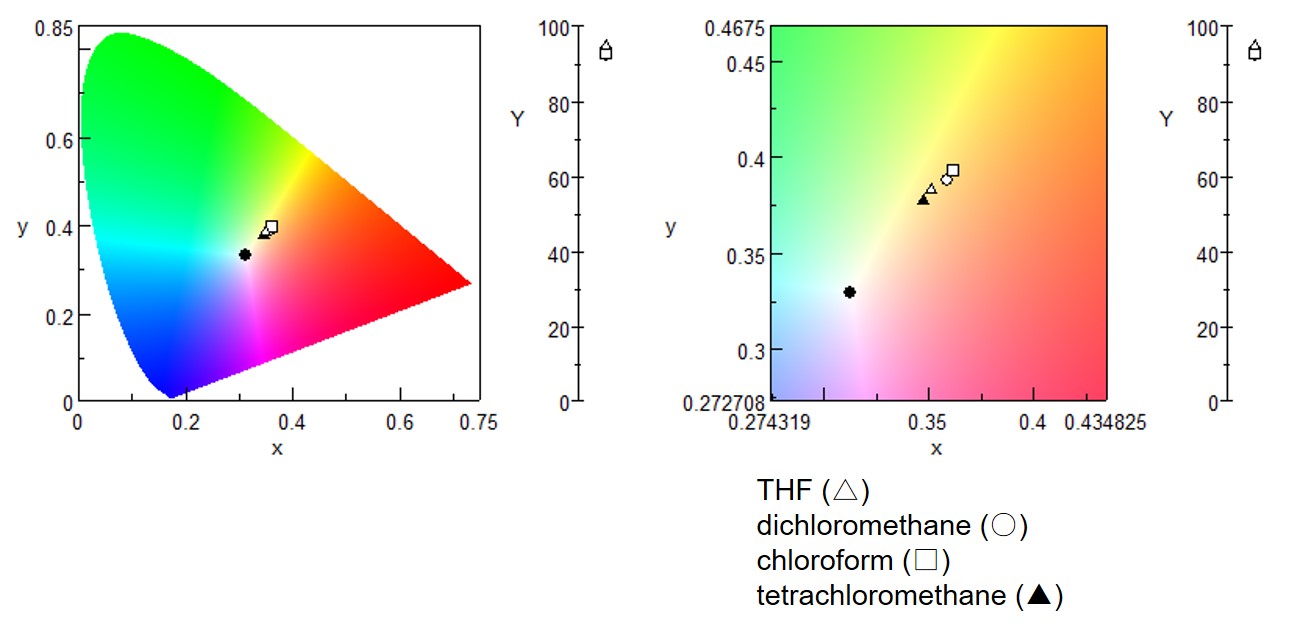


**Figure S9**. CIE colour space of PTh-*b*-PPI solution in tetrahydrofuran (THF), dichloromethane, chloroform and tetrachloromethane (concentration: 0.02 mg/ml).


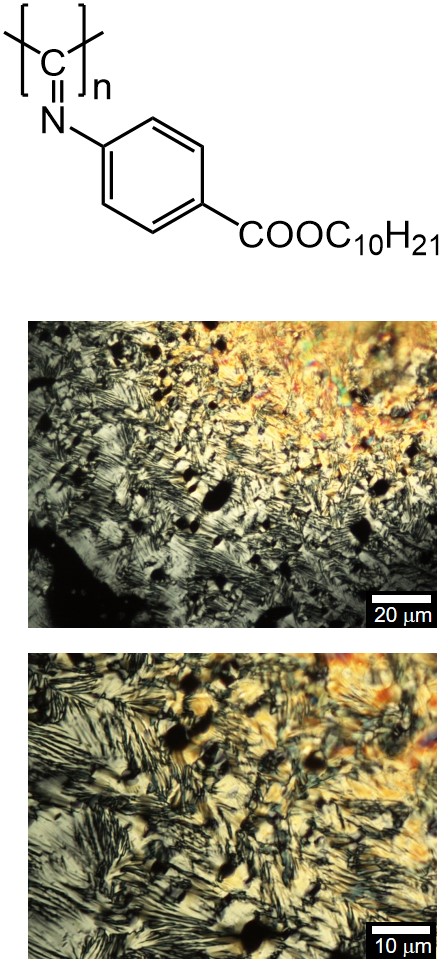


**Figure S10**. POM images of poly(decyl-phenylisocyanide) films on glass substrate at different magnification.

**References**

[1] R. Abbel, M. Wolffs, R. A. A. Bovee, J. L. J. Dongen, X.Lou, O. Henze, W. J. Feast, E. W.Meijer, A. P. H. J. Schenning, *Adv. Mater.*, **2009**, *21*, 597-602.

[2] R. S. Loewe, P. C. Ewbank, J. Liu, L. Zhai, R. D. McCullough, *Macromolecules*, **2001**, *34*, 4324–4333.

[3] Z.-Q. Wu, R. J. Ono, Z. Chen, C. W. Bielawski, *J. Am. Chem. Soc.* **2010**, 132, 14000–14001*.*

[4] a) H. Goto, E. Yashima, J. Am. Chem. Soc. 2002, 124, 7943–7949; b) G. Koeckelberghs, L. D. Cremer, W. Vanormelingen, T. Verbiest, A. Persoons, C. Samyn, Macromolecules, 2005, 38, 4545–4547.

[5] H. Goto, Adv. Funct. Mater. 2009, 19, 1335-1342.

[6] C. R. G. Grenier, S. J. George, T. J. Joncheray, E. W. Meijer, J. R. Reynolds, J. Am. Chem. Soc. 2007, 129, 10694–10699.

[7] T. Iseki, K. Kawabata, H. Kawashima, H. Goto, Polymer. 2014, 55, 66–72.
